# Wax On, Wax Off: Nest Soil Facilitates Indirect Transfer of Recognition Cues between Ant Nestmates

**DOI:** 10.1371/journal.pone.0019435

**Published:** 2011-04-29

**Authors:** Nick Bos, Lena Grinsted, Luke Holman

**Affiliations:** 1 Department of Biology, University of Copenhagen, Copenhagen, Denmark; 2 Department of Biological Sciences, Aarhus University, Aarhus, Denmark; Field Museum of Natural History, United States of America

## Abstract

Social animals use recognition cues to discriminate between group members and non-members. These recognition cues may be conceptualized as a label, which is compared to a neural representation of acceptable cue combinations termed the template. In ants and other social insects, the label consists of a waxy layer of colony-specific hydrocarbons on the body surface. Genetic and environmental differences between colony members may confound recognition and social cohesion, so many species perform behaviors that homogenize the odor label, such as mouth-to-mouth feeding and allogrooming. Here, we test for another mechanism of cue exchange: indirect transfer of cuticular hydrocarbons via the nest material. Using a combination of chemical analysis and behavioral experiments with *Camponotus aethiops* ants, we show that nest soil indirectly transfers hydrocarbons between ants and affects recognition behavior. We also found evidence that olfactory cues on the nest soil influence nestmate recognition, but this effect was not observed in all colonies. These results demonstrate that cuticular hydrocarbons deposited on the nest soil are important in creating uniformity in the odor label and may also contribute to the template.

## Introduction

Cooperation is predicted to evolve more readily when cooperators can assort with one another and exclude non-cooperators [1], [2], [3]. In many taxa, cooperation is preferentially directed towards kin or members of the same social group because these individuals have a higher probability of sharing cooperative genes and/or reciprocating the behaviour in the future. The evolution of social behaviour is therefore tightly intertwined with that of the recognition systems used to identify kin and group members.

Recognition of group members and non-members may be conceptualised as the comparison of a “label” to a “template” [4]. The label represents the combination of recognition cues borne by an individual or group, and could be composed of odours [5], cell surface receptors [6], colour markings [7] or other cues that provide information on identity. The template is a neural representation of the acceptable multivariate distribution of cues; a sufficiently large disparity between the label and template will lead to behavioural rejection of the individual carrying the label. The template may be immutable, for example because it is genetically encoded (as in “green beard” recognition [8]) or because it is imprinted at an early age without subsequent updating [9], [10]. However, in many taxa the template is plastic and is continuously updated. Most social insect species recognise non-nestmate individuals using a label composed of colony-specific odours carried on the body surface [5]. Experimental manipulation of these odours has been shown to cause the colony to update its template [11], [12], [13], [14], and honey bee (*Apis mellifera*) colonies have been found to adjust the breadth of the template with the frequency with which intruders are encountered and the costs of recognition errors [15], [16].

Within-group variation in the label confounds discrimination between group members and non-members by increasing the potential for overlap with the labels of other groups [5], [17]. Selection is therefore predicted to favour traits that homogenise recognition cues within groups. Social insects are thought to exchange odour cues with their nestmates by direct contact (e.g. grooming) and mouth-to-mouth food sharing (trophallaxis) [18]. Comparisons of genetically homogenous and heterogeneous ant colonies [19] and cross-fostering experiments [20] have shown that such cue-sharing can result in a highly uniform, colony-specific odour profile representing a combination of the individual odours present in the colony, termed the “Gestalt” [21]. As well as reducing the potential for recognition errors by minimising overlap between colonies' odour profiles, within-colony odour sharing may preclude within-colony conflicts and nepotism by mixing the cues that provide information on genetic identity [20], [22], [23].

Nestmate recognition in ants is based primarily on a class of chemicals called cuticular hydrocarbons (CHCs) [24], [25], [26], [27], [28], [29], [30]. Ants are thought to learn the CHC profile of their colony and continuously update their template by a process of habituation, such that novel combinations of CHCs trigger a rejection response [13], [14], [28]. CHC production has a substantial genetic component and therefore varies within genetically diverse colonies [20], [23], and ants exchange CHCs by trophallaxis [18], [31]. Experimental application of CHCs to ants or the substrate affects recognition [32], [33], [34] and CHCs have been detected on nest material [35], [36]. Furthermore, in *Polistes metricus* wasps, behavioural experiments have suggested that odour cues on the nest help to form the colony odour and influence the wasps' perception of the colony odour [37]. We therefore hypothesise that nest-borne CHCs may play an important role in shaping the recognition label, the template or both. However, definitive evidence that nest-borne CHCs function in nestmate recognition is lacking.

Here, we experimentally test whether nest soil affects the odour label and template in a soil-nesting ant species, *Camponotus aethiops*. We first determine whether contact with another colony's soil increases acceptance by that colony (i.e. nest soil affects the label), and evaluate whether the observed behavioural responses can be explained by indirect transfer of CHCs between ants via the soil. We then test whether ants exposed to soil from another colony become more likely to accept ants from that colony (i.e. nest soil changes the template). Our results suggest that indirect transfer of CHCs via the soil has an important role in homogenising the colony's odour label, and provide some evidence that odours present on nest material contribute to template formation.

## Materials and Methods

### Ant collection and culturing

Mature, queen-right *C. aethiops* colonies were collected near Castel del Rio (44°12′N 11°30′E), Italy, in April 2009. We collected soil along with the ants, selecting only the walls of major chambers and tunnels. Ant colonies were kept at room temperature in plastic boxes containing soil from their nest for 24–72 h before being used in experiments, to allow habituation and construction of tunnels and chambers in the collected soil. Colonies were provided with water, honey and dead insects *ad libitum*.

### Exposure to nest soil and aggression test protocols

To investigate whether nest soil plays a role in nestmate recognition, we conducted aggression tests involving medium-sized workers that had been exposed to soil from either their own colony or a different colony. Ants were placed, in pairs, in plastic coffee cups (diameter 45 mm) with Fluon-coated sides containing approximately 10 ml of nest soil and a ball of moist cotton. Ants were kept in the cups for 24 h prior to use in aggression tests, and cups were not re-used.

Aggression tests took place in a plastic Petri dish arena (100 mm×15 mm) with Fluon-coated sides and a filter paper floor (replaced between trials). The “target” ant was freeze-killed, allowed to warm to room temperature, then placed in the centre of the arena surrounded by a plastic barrier using pentane-cleaned forceps. The “focal” ant was placed in the arena for at least 1 minute to habituate, before the barrier around the target ant was removed, starting the test. During the 180 s observation period, we used Etholog 2.2.5 software [38] to record the duration of the following four behaviours by the focal ant: antennation, mandible opening, biting and gaster flexing (i.e. potentially spraying formic acid). The behavioural data were used to calculate an aggression index as in [39], which ranged from 1–4. The aggression tests were performed by two observers, who conducted an equal number of tests for all colony/treatment combinations (the random factor “observer” did not explain a significant proportion of the residual deviance, and was not used in the final analyses). All data recording was blind with respect to treatment.

For both experiments, we used five pairs of colonies; the experimental design was fully independent, such that one colony in each pair always provided the focal ants and one the target ants. Individual ants were only used once. We pre-screened all colony pairs to ensure that they were aggressive towards one another. Aggression tests in which the focal ant did not contact the target ant during the three minute trial were excluded from the analysis (n = 62 tests).

### Experiment 1: Does nest soil affect the odour label?

To test whether exposure to nest soil changes an ant's chemical profile (its label) and affects the likelihood of being aggressively rejected by conspecifics, target ants were exposed to soil from either their own colony (the control; n = 82), or the same colony as the focal ant (n = 79), and used in aggression tests. The focal ants were removed from their home colonies immediately before the aggression tests.

### Experiment 2: Does nest soil affect the odour template?

To test whether exposure to nest material influences an ant's perception of its own colony odour (the template) and therefore its propensity to reject ants from other colonies, focal ants were exposed to soil from either their own colony (the control; n = 70), or from the same colony as the target ant (n = 68), prior to use in aggression tests. Target ants were removed from their home colonies and freeze-killed immediately before the test.

### Cuticular hydrocarbon analysis

We analysed the CHCs of 9–10 ants from each of the 20 combinations of colony and soil treatment (n = 196). Gas chromatography-mass spectrometry (GC-MS) was performed as described in [39]. Table 1 lists the hydrocarbon peaks used in the present study.

**Table 1:** List of cuticular hydrocarbons found on *Camponotus aethiops*.

Peak	Identity	%	SE	Transmission index	SE	Diagnostic power
1	C_23_	0.34	0.01	0.11	0.14	1.70
2	2-MeC_24_	1.09	0.04	3.47	3.45	2.27
3	C_25_	1.23	0.04	0.01	0.15	1.58
4	13-, 11- & 9-MeC_25_	3.11	0.06	0.59	0.47	3.02
5	7-MeC_25_	1.55	0.07	0.06	0.05	5.55
6	5-MeC_25_	0.41	0.01	−0.08	0.21	2.51
7	11,15- & 9,13-diMeC_25_	2.36	0.09	15.59	14.62	2.80
8	7,9- & 7,11- & 7,13- & 7,15-diMeC_25_ and 3-MeC_25_	1.89	0.06	0.02	0.13	1.68
9	5,9- & 5,13-diMeC_25_	0.45	0.02	0.01	0.06	3.16
10	5,17-diMeC_25_	0.53	0.03	0.03	0.08	3.23
11	C_26_	1.02	0.04	0.26	0.14	1.51
12	3,13-, 3,11-, 3,9- & 3,7-diMeC_25_	2.76	0.11	−0.01	0.03	4.90
13	13- & 12-MeC_26_	1.36	0.04	0.59	0.60	2.04
14	8-MeC_26_ & x,y-diMeC_26_	3.10	0.12	−0.17	0.13	2.12
15	6-MeC_26_	0.27	0.03	−0.14	0.19	1.03
16	4- & 2-MeC_26_	7.87	0.20	4.77	4.16	1.77
17	C_27_	3.39	0.13	0.54	0.30	1.15
18	13- & 11-MeC_27_	10.37	0.21	0.53	0.50	2.10
19	9-MeC_27_	4.36	0.05	5.70	4.22	1.27
20	7-MeC_27_	4.23	0.09	0.14	0.09	1.79
21	5-MeC_27_	2.87	0.12	0.58	0.73	1.63
22	11,15-diMeC_27_	8.16	0.23	0.62	0.45	2.79
23	9,13-diMeC_27_	4.84	0.10	−0.14	0.18	1.69
24	7,15-, 7,13-, 7,11-diMeC_27_ & 3-MeC_27_	3.31	0.04	0.35	0.29	1.48
25	5,7-, 5,9-, 5,13-, 5,15- & 5,17-diMeC_27_	3.34	0.06	0.08	0.11	1.29
26	C_28_	0.63	0.03	0.92	0.42	1.45
27	3,15- & 3,13- & 3,9- & 3,7-diMeC_27_	6.33	0.27	0.13	0.09	3.95
28	14-, 13-, 12-, 10-, 8- & 6-MeC_28_	4.03	0.05	0.28	0.23	1.75
29	12,16-diMeC_28_ & 4-MeC_28_	1.61	0.04	0.09	0.11	2.22
30	C_29_	0.69	0.03	0.22	0.21	1.32
31	15- & 13- & 11- & 9-MeC_29_	3.90	0.11	−0.11	0.05	3.29
32	7-MeC_29_	1.88	0.04	0.03	0.14	1.88
33	5-MeC_29_	0.49	0.02	0.00	0.13	2.78
34	13,17-, 11,15- & 9,13-diMeC_29_	1.96	0.15	0.12	0.13	5.00
35	7,17-diMeC_29_ & 3-MeC_29_	1.97	0.03	0.19	0.19	1.66
36	5,17-diMeC_29_	2.30	0.09	0.07	0.04	4.11

The table shows the percentage in the profile (mean and SE), the transmission index (mean and SE) and the diagnostic power of each peak (n = 196 ants from ten colonies).

To assess whether soil treatment made ants' chemical profiles become more similar to ants from the colony providing the soil, we calculated a “transmission index” for each CHC as follows:
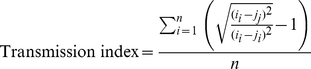
(1)where *i_i_* is the mean proportion of the focal CHC in ants from the *i*th colony that had been housed in *i*th colony soil, *j_i_* and *j_j_* and are the corresponding values for the paired colony *j* after being treated with *i* or *j* soil respectively, and *n* is the number of colonies (i.e. ten). The transmission index describes the ratio of chemical similarity between A) the focal colony and its paired colony when the paired colony was exposed to its own soil, and B) the focal colony and the paired colony when the paired colony was exposed to focal colony soil. The index is positive if the proportion of the hydrocarbon became more similar to the value of the colony providing the soil, negative if it became more dissimilar and zero if soil treatment had no effect on the difference between the colonies. We also calculated the diagnostic power of each CHC as described in [39]; peaks with high diagnostic power have low variability within colonies relative to between colonies, and therefore provide more information on an individual's colony of origin. All analyses were performed in R 2.8.1; all GLMMs were calculated using the Laplace approximation implemented in the lme4 package for R, and use a quasipoisson error structure to account for non-normal errors and overdispersion.

## Results

### Effect of soil treatment on non-nestmate recognition

**Figure 1:**
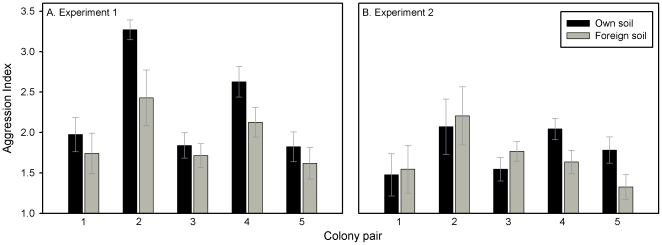
Testing the effect of nest soil on the olfactory label and template of *C. aethiops* carpenter ants. A) Exposing target ants to soil from another colony caused them to receive significantly less aggression from ants from the colony supplying the soil, demonstrating that nest soil contributes to the odour label (n = 161). B) Exposing a focal ant to soil from another colony had no consistent effect on its aggressive response to ants from the colony supplying the soil. The aggression level of treated ants did not differ from controls in colony pairs 1, 2 and 3, but there was a significant effect in pair 5 and a non-significant trend in pair 4 for lowered aggression in focal ants exposed to the paired colony's soil (n = 138).

In experiment 1, we found a significant effect of soil treatment on aggression. Aggression was lower when the target ant had been exposed to nest soil from the focal ant's colony, relative to the control (Figure 1A; GLMM with colony pair as a random factor: t_155_ = 2.75, p = 0.007). The direction of this effect was consistent across all five colony pairs (Figure 1A). By contrast, in experiment 2, focal ants that had been exposed to the nest material of the target ant were not significantly less aggressive towards the target relative to controls across all five colony pairs (Figure 1B; GLMM: t_132_ = 1.07, p = 0.29). However, in colony pairs 4 and 5, controls were less aggressive; this difference was significant in colony pair 4 (GLM: t_40_ = 2.02, p = 0.04) and marginally non-significant in pair 5 (GLM: t_26_ = 1.96, p = 0.06). We also recorded higher levels of aggression in experiment 1 than in experiment 2 (Figure 1).

### Effect of soil treatment on cuticular hydrocarbons

The transmission index was positive for 29/36 hydrocarbons, a significantly higher number than predicted if soil treatment had no effect on ants' CHCs (two-tailed binomial test: p = 0.005; simulated 95% CIs = 12–24). Additionally, the transmission index of a peak was non-significantly, positively correlated with the proportion of the chemical profile composed of each compound (Spearman's ρ = 0.32, p = 0.057). This result suggests that the major components of the CHC profile may be more easily transferred from ants to the nest soil, and from there onto another ant. The median transmission index of the 36 peaks was 0.13 (Table 1; range: −0.17–15.6), indicating that 24 h of exposure to allocolonial soil caused the relative abundance of each CHC to become around 13% more similar to that of ants from the colony providing the soil.

In addition to the convergence of odours that occurred between colonies following treatment with one another's soil, we also found that non-nestmate soil induced a non-colony-specific change in the chemical profiles of treated ants. Ants that had been housed in non-nestmate soil had a significantly higher proportion of three CHC peaks in their profile than did ants housed in own-colony soil. The peaks 5,9- & 5,13-diMeC_25_, 8-MeC_26_ & x,y-diMeC_26_ and 5,17-diMeC_29_ were always higher in the non-nestmate soil-treated ants, regardless of the proportions of those chemicals in the non-nestmate colony relative to the focal colony (all p<0.02; GLMMs with ant colony and soil colony as random factors; peaks 9, 14 and 36 in Table 1).

There was no relationship between diagnostic power and the transmission index (Spearman's ρ = 9480, p = 0.20). The diagnostic power of linear alkanes was significantly lower than that of methylalkanes (GLM: t_33_ = 2.27, p = 0.03) and dimethylalkanes (t_34_ = 3.08, p = 0.004), suggesting that linear alkanes encode the least amount of information about colony identity. There was no correlation between the size of a peak and its diagnostic power (ρ = 7150, p = 0.64), and no relationship between the type of cuticular hydrocarbon (linear, methylalkane or dimethylalkane) and the transmission index (GLM: F_2,33_ = 0.44, p = 0.65).

We also tested for a relationship between chemical distance (as measured by Euclidean distance) and the aggression index across the 20 combinations of treatment, colony and experiment, and found no significant effect (GLMM with colony pair and experiment as random factors; t = −0.63, n = 20, p>0.1).

## Discussion

Our results demonstrate that the odour profile of ants is influenced by the nest soil in which they live, and that this change in odour significantly affects non-nestmate recognition and aggression. The cuticular hydrocarbons (CHCs) of ants treated with foreign colony soil became more similar to the CHCs of ants from the foreign colony, showing that nest soil can transfer CHCs from ant to ant. The observed convergence of ants' profiles following soil treatment was small for some peaks, but we believe that the effect would be greater in nature, where ants are in near-constant contact with nest soil (our experimental exposures lasted 24 h). Given the importance of CHCs to nestmate recognition in ants, it is likely that CHC transfer via the soil explains the reduced aggression observed towards non-nestmate ants treated with soil from the focal ant's colony (Experiment 1), although it is possible that odours other than CHCs were also involved. Our results suggest that the hydrocarbons deposited on soil are colony-specific in *C. aethiops*, in contrast to a recent study of the ant *Lasius niger*, which sampled CHCs on the soil directly and did not find a difference among colonies [36].

We found some evidence that ants exposed to a foreign colony's soil updated their odour template and became less aggressive towards members of that colony, but this effect did not occur in all five colony pairs. However, previous work on this species [14] and the congeneric *C. herculeanus* [13] has shown that ants exposed to novel CHCs do update their templates, implying that non-nestmate recognition represents sensory adaptation or habituation to frequently-encountered odours [5]. One potential explanation for this discrepancy is that the concentration of CHCs on nest soil is not high enough to significantly affect the template, at least over the 24 h timescale we employed. Another possibility is that the lower overall aggression levels observed in soil-treated focal ants (Experiment 2), which may have been caused by the ants' 24 h of isolation [40], masked differences in perception. In other words, the ants could perceive the odour differences but did little to act on them; disentangling the action and perception components of recognition is a recurring challenge in recognition studies [41], [42].

As well as facilitating the transfer of ant-derived CHCs, exposure to foreign soil caused significant increases in the amount of three specific CHC peaks in all treated ants. Because the three CHC peaks always increased, irrespective of their abundance in the profiles of the treated colony and the colony providing the soil, we suggest that the stimulus of soil containing non-nestmate cues and/or the absence of cues associated with the home colony might cause a physiological change in the treated ants, resulting in increased production of these hydrocarbons. Rapid changes in ants' CHC profiles have been recorded previously after immune challenge [43] and mating [44]; the changes may reflect altered CHC synthesis or transport to the cuticle, or modified grooming behaviour.

We found that the diagnostic power of linear alkanes was significantly lower than that of methylalkanes and dimethylalkanes, implying that the former provide less information about colony identity (see also [13], [20], [29], [39]). We found no correlation between the size of a peak and its diagnostic power, suggesting that both major and minor components of the chemical profile may be involved in non-nestmate recognition. No correlation was found between the diagnostic power and transmission index of cuticular hydrocarbons, suggesting that the degree to which CHCs are transferred via the soil is unrelated to the information they provide regarding colony identity.

Some social parasites obtain recognition cues from their hosts in order to avoid detection, termed chemical camouflage [45], [46]. Our results suggest a potentially widespread mechanism by which social parasites of ants may acquire host chemical cues through contact with nest material. Our findings parallel a behavioural study of the socially parasitic wasp *Sulcopolistes sulcifer*, which apparently obtains the colony odour of its host *Polistes dominulus* by rubbing its abdomen against the nest comb [47].

In summary, we show that cuticular hydrocarbons can be transferred from one ant to another via the nest soil, and that this transfer affects non-nestmate recognition. Along with allogrooming and trophallaxis, indirect exchange via the nest material contributes to the formation of a uniform colony odour, potentially reducing errors in nestmate recognition and minimising the opportunity for within-colony nepotism. Transfer of recognition cues among nestmates via the soil may be adaptive, and selection for ease of transmission may have shaped the evolution of the cuticular hydrocarbon profile.
